# The influence of single-point mutation D614G on the binding process between human angiotensin-converting enzyme 2 and the SARS-CoV-2 spike protein-an atomistic simulation study†

**DOI:** 10.1039/d3ra00198a

**Published:** 2023-03-28

**Authors:** Chengcheng Shi, Yanqi Jiao, Chao Yang, Yao Sun

**Affiliations:** a School of Science, Harbin Institute of Technology (Shenzhen) Shenzhen 518055 China sunyao0819@hit.edu.cn; b State Key Lab of Urban Water Resource and Environment, School of Science, Harbin Institute of Technology (Shenzhen) Shenzhen 518055 China xyyang@hit.edu.cn

## Abstract

SARS-CoV-2 has continuously evolved as changes in the genetic code occur during replication of the genome, with some of the mutations leading to higher transmission among human beings. The spike aspartic acid-614 to glycine (D614G) substitution in the spike represents a “more transmissible form of SARS-CoV-2” and occurs in all SARS-CoV-2 mutants. However, the underlying mechanism of the D614G substitution in virus infectivity has remained unclear. In this paper, we adopt molecular simulations to study the contact processes of the D614G mutant and wild-type (WT) spikes with hACE2. The interaction areas with hACE2 for the two spikes are completely different by visualizing the whole binding processes. The D614G mutant spike moves towards the hACE2 faster than the WT spike. We have also found that the receptor-binding domain (RBD) and N-terminal domain (NTD) of the D614G mutant extend more outwards than those of the WT spike. By analyzing the distances between the spikes and hACE2, the changes of number of hydrogen bonds and interaction energy, we suggest that the increased infectivity of the D614G mutant is not possibly related to the binding strength, but to the binding velocity and conformational change of the mutant spike. This work reveals the impact of D614G substitution on the infectivity of the SARS-CoV-2, and hopefully could provide a rational explanation of interaction mechanisms for all the SARS-CoV-2 mutants.

## Abstract 2

SARS-CoV-2 has continuously evolved as changes in the genetic code occur during replication of the genome, with some of the mutations leading to higher transmission among human beings.

## Graphical abstract

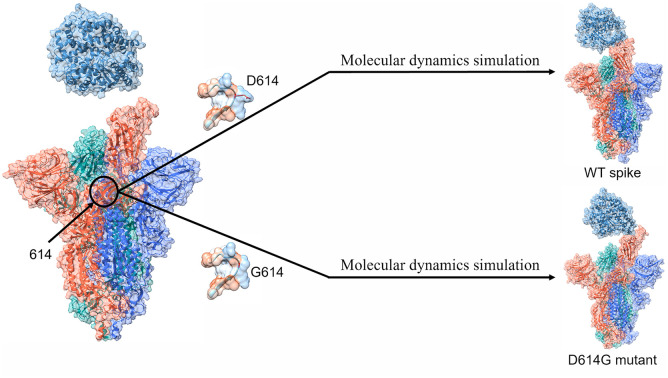

## Introduction

**Fig. 1:**
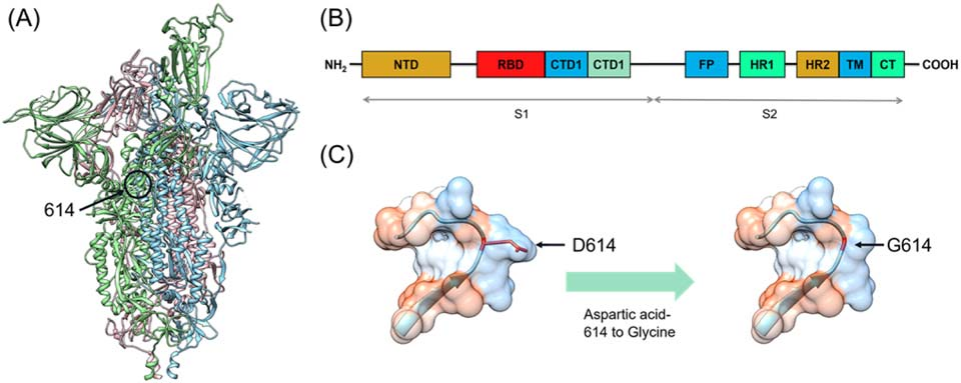
(A) Overall structure of the SARS-CoV-2. The three monomers are colored in blue, green and pink. (B) Schematics of the SARS-CoV-2 spike monomer. The NTD, RBD, CTD1, CTD2, FP, HR1, HR2, TM, and CT denote the N-terminal domain, receptor-binding domain, fusion peptide, heptad repeat 1, heptad repeat 2, transmembrane domain, and cytoplasmic tail respectively. (C) The spike with aspartic acid-614 to glycine (D614G) mutation. The red color indicates mutation of amino acid 614.

The COVID-19 is caused by the severe acute respiratory syndrome coronavirus 2 (SARS-CoV-2) which is a positive RNA virus that could lead to severe respiratory syndrome in human beings.^1^ Effective interventions such as vaccines, antibodies, and inhibitors are always needed for the control of a worldwide epidemic. The SARS-CoV-2 genome encodes 16 non-structural proteins (nsp1–16) and four structural proteins, including the spike (S), nucleocapsid (N), membrane (M), and envelope (E).^2^ The spike comprises three identical protomers (Fig. 1A), which is totally composed of 1273 amino acids, including a signal peptide (amino acids 1–13) located at the N-terminus, a S1 subunit (amino acids 14–685) and a S2 subunit (amino acids 686–1273). The S1 and S2 subunits are mainly responsible for receptor binding and membrane fusion, respectively. The S1 subunit can be further divided into the N-terminal domain (NTD amino acids 14–305), receptor-binding domain (RBD, amino acids 331–527) and C-terminal domains 1 and 2 (CTD1 528–590 and CTD2 591–685) (Fig. 1B). The S2 subunit comprises the fusion peptide (FP) (amino acids 788–806), heptapeptide repeat sequence 1 (HR1) (amino acids 912–984), HR2 (amino acids 1163–1213), transmembrane domain (TM) (amino acids 1213–1237), and cytoplasmic tail (CT) (amino acids 1237–1273)^3^ (Fig. 1B). The infectious ability of the SARS-CoV-2 is related to the spike, which invades cells by interacting with human angiotensin-converting enzyme (hACE2). When attacking host cells, RBD in the S1 subunit binds with hACE2 on the cell surface.^4–6^ NTD works in two ways. The first is to work with RBD through the interactions with co-factors L-SIGN and DC-SIGN on the cell surface.^7^ The other is to keep spike in the closed position by interacting with RBD.^8^ Subsequently, the S1 and S2 subunits are divided by furin-like pro-protein convertase (PCs), resulting in the dissociation of S1 and the irreversible refolding of S2 into a post-fusion structure.^9,10^ Such process induces fusion of the SARS-CoV-2 and the host cell membranes.

Fortunately, most of the mutants have not yet broken out as worldwide pandemic though the SARS-CoV-2 has mutated thousands of times.^11^ Five among the mutants have caused severe concern, which are B.1.1.7 (Alpha), B.1.351 (Beta), B.1.1.28.1 (Gamma), B.1.617.2 (Delta), and B.1.1.529 (Omicron). Alpha mutant was firstly discovered in the UK in September 2020 and quickly spread to other countries.^12^ There are totally 17 mutations in the spike of Alpha mutant, containing two key nonsynonymous mutations, *i.e.*, N501Y and D614G. At the same time, the South African mutant Beta was discovered, which includes K417N, E484K, and N501Y mutations in RBD of the spike as well as the D614G mutation.^13,14^ Gamma mutant strain was later reported in Brazil in December 2020, in which RBD contains N501Y, E484K, and K417T mutations. It should be noted that D614G mutation also appears in the Gamma mutant spike.^15^ In March 2021, the Delta mutation strain broke out in India and has aroused fear because of its increased infectivity, high virulence ability, and potential immune escape. Delta contains two mutations (L452R and T478K) in RBD and the D614G mutation in the spike.^16–18^ In the latest November 2021, the newest mutant strain named Omicron appeared, which has been reported to contain the twice number of mutations than that of the Delta. Omicron shows 15 mutation sites in RBD of the spike, and its propagation speed remains unknown.^19^ At present, many research works have been focused on the influences of relevant mutations on the interaction between the spike and hACE2 through experiments and calculations. David and co-worker have used atomic force microscopy and molecular dynamics (MD) simulation to test the equilibrium dissociation constants (*K*_D_) and interaction energy of the wild-type (WT), alpha, beta, kappa and gamma with hACE2.^20^ Khan and co-workers have studied the effects of mutated spike on binding sites and strength with hACE2 through MD simulations.^21^ It is worthy of noting that the D614G mutation exists in the spikes (Fig. 1C) of all the five mutants mentioned above.^22^ Researchers have shown that patients infected with the D614G mutants have higher viral loads, proving their higher transmission, infectivity and replication rate.^23,24^ Structural analysis has reveals that RBD of the D614G mutant occupies a higher percentage in the open conformation than the WT spike, which means that the D614G mutant possesses improved ability to bind to hACE2.^25^ However, the exact impact of the D614G mutation on the interaction between the mutant spike with the human cells is not fully understood.^26^ In addition, how the D614G mutation alters the conformation of spike-hACE2 complex has been rarely studied. Therefore, investigating the binding behavior and interaction between the D614G mutant spike and hACE2 together with the conformation of their complex are of significance towards a deep understanding of mutation induced binding affinity/infectivity change and could hopefully offer some guidance on development of effective treatment.

The commonly adopted way to study the protein structure and interaction between the spike and hACE2 is through the cryo-electron microscopy (Cryo-EM).^27,28^ However, the Cryo-EM could not reveal the overall binding processes of the two, let alone the high testing costs. As the early appeared SARS-CoV-2 D614G mutant, the D614G mutation has drawn attention of many researchers. Some researchers have investigated the impact of D614G mutation on the spike by MD simulations.^29–31^ For example, Gnanakaran and co-worker have found the open conformational states of D614G mutant spike by MD simulations and proposed that the increased infectivity is likely to be associated with conformational changes and/or an increase in the population of open states.^32^ Andricioae and co-worker have used MD simulation, tICA analysis, and mutual information-based network to explore the mechanism of D614G as the distant modulation of conformational open of the spike.^33^ Samuel and co-worker have reported that the different conformational stabilities of WT and D614G mutant spikes are mainly driven by the high displacements of backbone atoms in the S2-domain.^34^ However, the current studies have not referred to the detailed contact process of D614G mutant spike with hACE2. In addition, the experimental resolved structures of D614G mutant spike in the open protein database lack certain fragments, so that the simulation works based on these public structures are not reasonably accurate. In this paper, we used the deep learning framework AlphaFold2 (Google, DeepMind)^35^ to construct our single-point mutation D614G spike and conducted the MD simulations to reveal the dynamic binding process between hACE2 and the mutant spike, through which we could find out the initial interaction area, visualize the whole binding process and reveal the conformational changes of mutant spike-hACE2 complex. By comparing with the corresponding results of the WT spike with hACE2, the interaction characteristics due to the single-point mutation D614G could be well explained.

## Methods

### Preparation of the protein model

**Fig. 2:**
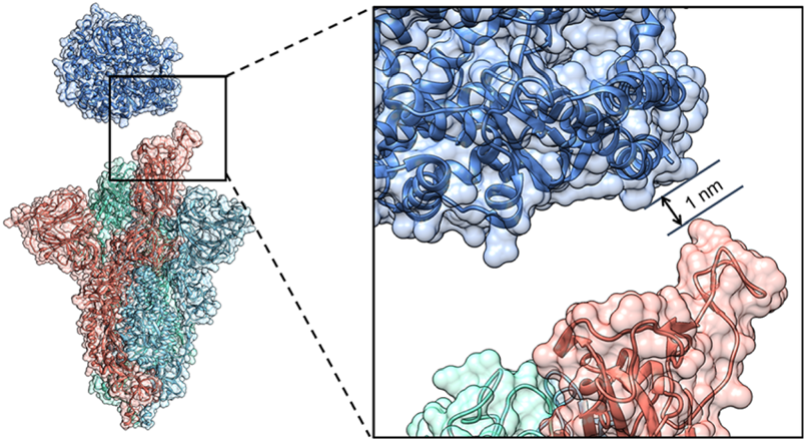
The initial structure of the WT spike-hACE2 complex. The distance between the spike and hACE2 is 1 nm. The B chain, C chain, D chain of the spike and hACE2 are colored in red, royal blue, cyan and steel blue respectively.

The Cryo-EM structure of the WT spike-hACE2 complex (Fig. 2, PDB ID: 7DF4)^27^ was obtained from the Protein Data Bank.^36^ Names and abbreviations of amino acids are shown in Table S1 in the ESI.† The distance between hACE2 and the spike was greater than 1 nm (10 Å). The WT spike-hACE2 complex structure was prepared by removal of water molecules and co-crystal ligands, leaving only the amino acid residues. The D614G mutant spike was created by using glycine to substitute for aspartic acid at position 614 of the WT spike with UCSF Chimera 1.15.^37^ Accordingly, we used AlphaFold2 and homologous modelling to predict the single-chain structural models of the D614G mutant spike. The predicted single-chain (B chain) structural model of D614G mutant spike by AlphaFold2 has the high pLDDT value of 0.8824 compared with the WT spike model (PDB ID: 7DF4, B chain). The two structural models have a RMSD comparison value of 1.502 Å (Fig. S1A in the ESI†). However, the single-chain (B chain) structural model of D614G mutant spike by homologous modelling has the overall quality factor of 62.332 compared with the WT spike model (B chain). The two structural models have a RMSD comparison value of 6.991 Å (Fig. S1B in the ESI†). Therefore, our D614G mutant spike model was generated based on the single-chain prediction by AlphaFold2.

### Molecular dynamics (MD) simulations

The AMBER99SB-ILDN force field^38^ was used to parameterize the proteins, as implemented in the GROMACS 5.1.2 (ref. 39) software package. The particle mesh Ewald (PME) method was used to calculate the long-range electrostatic interactions.^40,41^ Each simulation was performed in explicit water solvent through the TIP3P^42^ water box with dimensions of 27.58 × 27.58 × 27.58 nm^3^ and periodic boundary condition. Thirty-three (33) Na^+^ ions were introduced into the water box to neutralize the charge of the entire system. Energy minimization and equilibrations were carried out in three steps: (i) we minimized the whole system containing ions, solvent and proteins for up to 100 000 steps using a steepest-descent algorithm. (ii) The SARS-CoV-2 S trimer and hACE2 were equilibrated to 310 K [normal human temperature, NVT equilibration, 100 ps, V-rescale (the velocity rescale method)] with backbones restrained. (iii) NPT ensemble was used at constant pressure (1 bar) and temperature (310 K) with ACE2 restrained for 500 ps using a time step of 2 fs for the equilibration. We have found these papers for the SARS-COV-2 spike protein with MD simulations for 150–200 ns.^43–46^ Finally, the MD run was conducted for 150 ns with all the constraints released.

### Analyses of architectures and binding processes

**Fig. 3:**
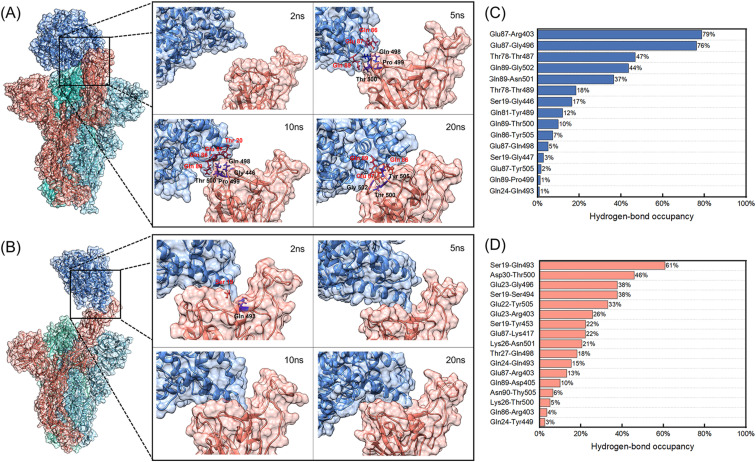
(A) and (B) Binding processes of the WT and D614G mutant spikes with hACE2 at 2 ns, 5 ns, 10 ns and 20 ns. The spike B chain, C chain, D chain and hACE2 are colored in red, royal blue, cyan and steel blue, respectively. (A) The WT spike with hACE2; (C) the D614G mutant spike with hACE2. The contact residues are marked in the images. (C) and (D) Hydrogen-bond occupancies of interacting residues between hACE2 and B chains of spikes during the 150 ns MD simulations. (C) The WT spike with hACE2; (D) the D614G mutant spike with hACE2.

**Fig. 4:**
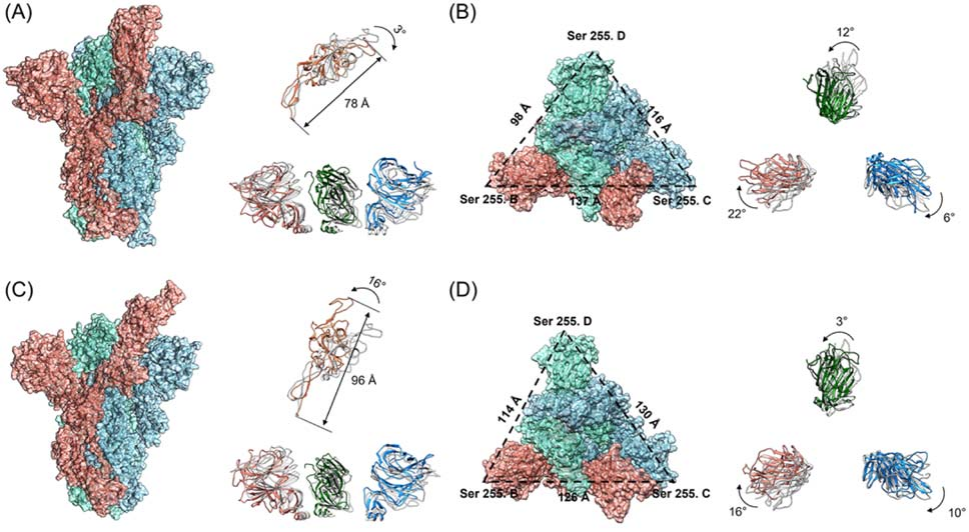
The architectures of the SARS-CoV-2 WT and D614G mutant spikes after the 150 ns MD simulations. The RBD and NTD structures of the spikes after the 150 ns MD simulations are colored while their structures before the simulation are in dark grey. The B chain, C chain and D chain are colored in red, blue and green respectively. (A) The side views of the WT spike, RBD and NTD structures; (B) the top views of the WT spike and NTD structures; (C) the side views of the D614G mutant spike, RBD and NTD structures; (D) the top views of the D614G mutant spike and the NTD structures.

**Fig. 5:**
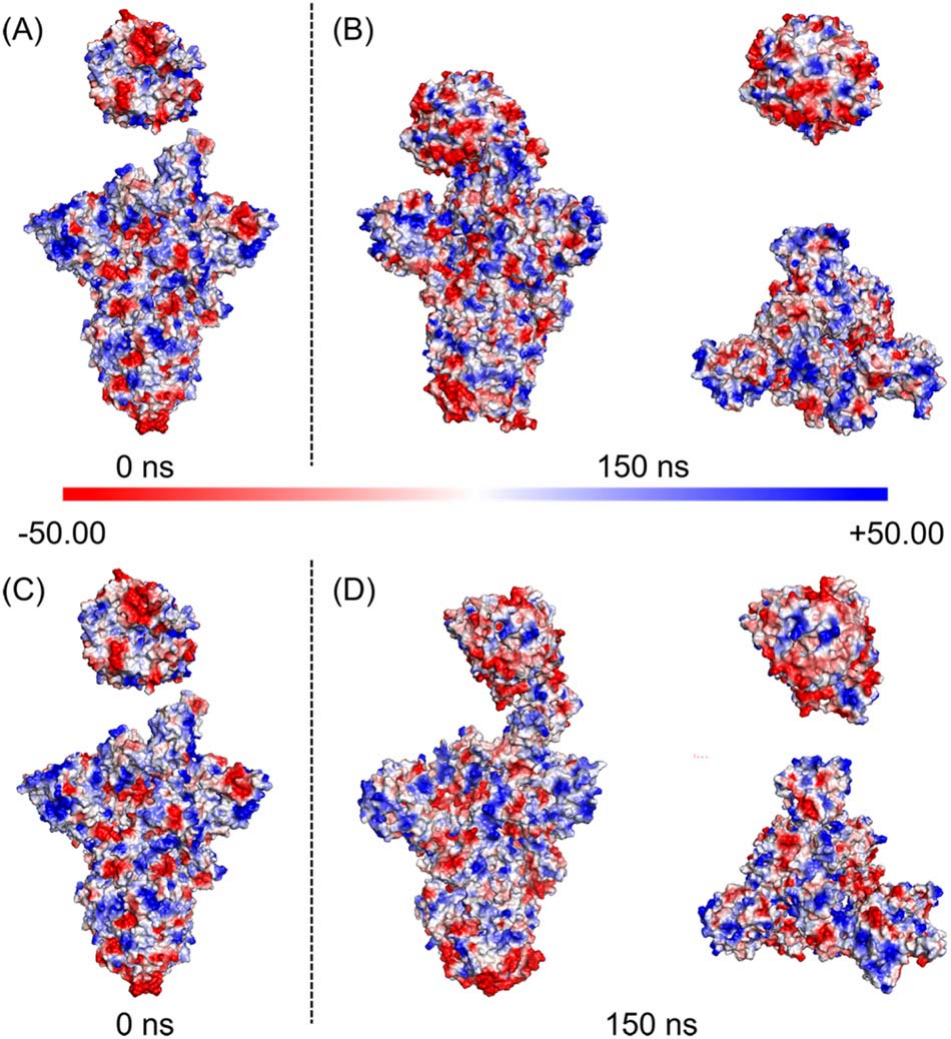
The electrostatic surface potentials of the SARS-CoV-2 WT and D614G mutant spikes with hACE2. Protein surface is colored according to the electrostatic potential. The scale of color bar ranges from −50 kT/e (red) to +50 kT/e (blue); (A) electrostatic surface potential of WT spike-hACE2 complex at 0 ns; (B) electrostatic surface potential of WT spike-hACE2 complex, hACE2 and top views of WT spike at 150 ns; (C) electrostatic surface potential of D614G mutant spike-hACE2 complex at 0 ns; (D) electrostatic surface potential of D614G mutant spike-hACE2 complex, hACE2 and top views of D614G mutant spike at 150 ns.

The PDBsum^47^ was used to generate the 2D visualizations, number of interface residues, interface areas, numbers of hydrogen bonds and non-bonded interactions (Table 1, Fig. S2 and S3 in the ESI†). Based on the results of PDBsum, the softwares VMD^48^ and UCSF ChimeraX 1.2.5 (ref. 49) were used to visualize the 3D binding processes of the D614G mutant and WT spikes with hACE2 (Fig. 3A and B). The UCSF Chimera 1.15 was adopted to analyse the molecular architectures of SARS-CoV-2 WT and D614G mutant spikes (Fig. 4). The PyMOL 2.5.2 (ref. 50) was used to analyse the electrostatic surface potentials (Fig. 5). Distances between residue centers of interaction of hACE2 and spike were generated through Go-Contact Map^51,52^ (Tables S2 and S3 in the ESI†).

**Table 1:** Number of interface residues, interface areas (Å^2^), number of hydrogen bonds and non-bonded interactions of the WT and D614G mutant spikes with hACE2 at different time

		Number of interface residues		Interface areas (Å^2^)		Number of hydrogen bonds	Number of non-bonded interactions
		hACE2	Spike	hACE2	Spike		
WT spike	2 ns	0	0	0	0	0	0
	5 ns	3	3	174	183	1	10
	10 ns	4	4	178	178	1	14
	20 ns	3	3	123	118	2	12
	50 ns	14	16	725	687	8	86
	75 ns	10	13	649	580	3	50
	100 ns	15	19	748	685	3	80
	150 ns	10	16	704	637	7	60
D614G mutant spike	2 ns	1	1	118	106	1	2
	5 ns	4	6	276	231	5	36
	10 ns	5	11	434	358	5	50
	20 ns	7	9	482	448	4	55
	50 ns	8	9	384	353	10	41
	75 ns	5	7	309	363	2	29
	100 ns	7	9	477	432	4	42
	150 ns	9	12	542	514	2	67

### Analyses of MD simulations

**Fig. 6:**
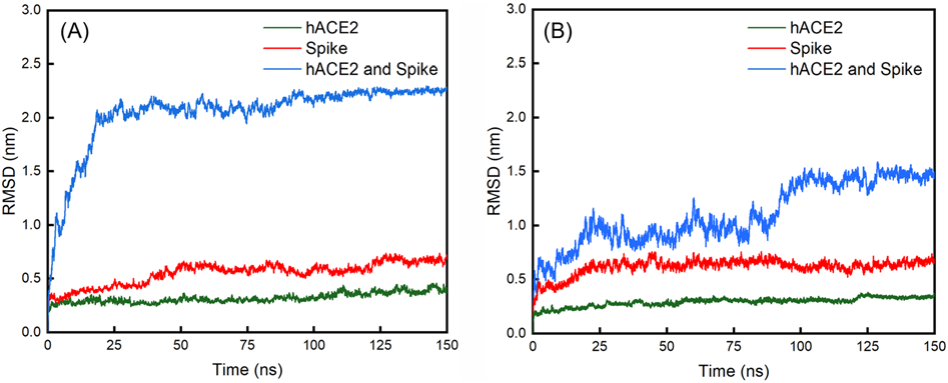
Evolutions of the RMSD values over 150 ns for the (A) WT spike, hACE2 and their complex; (B) D614G mutant spike, hACE2 and their complex.

**Fig. 7:**
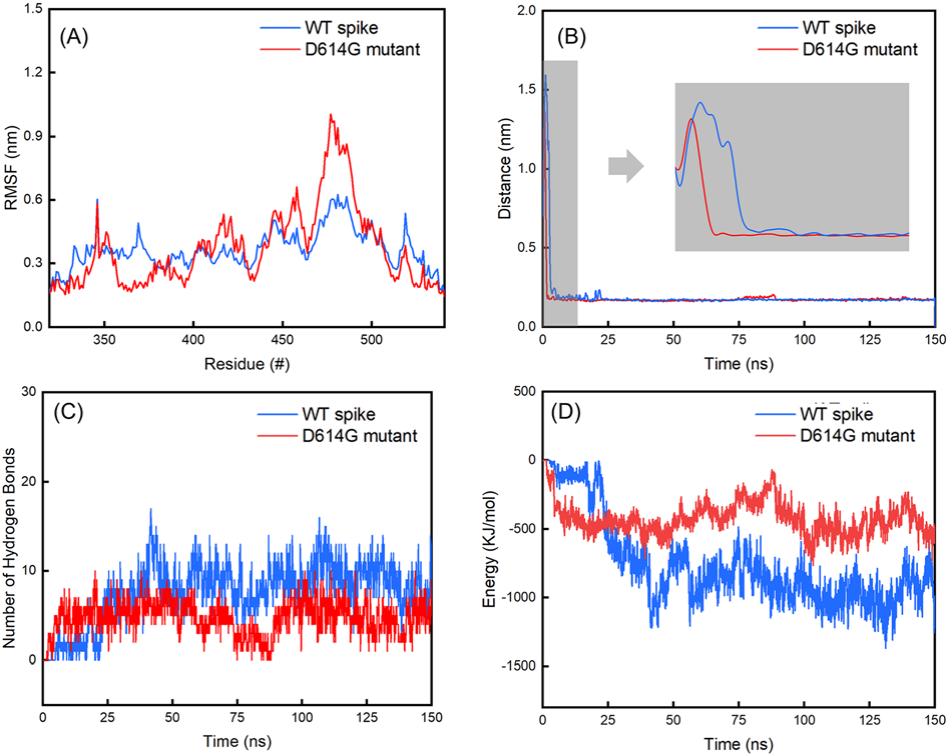
(A) Evolutions of the RMSF values of residues in the RBD regions of the B chains in spikes over the last 25 ns of 150 ns simulations; (B) distances between the spikes and hACE2; (C) number of hydrogen bonds between the spikes and hACE2; (D) interaction energy between the spikes and hACE2.

The hydrogen bond, hydrogen-bond occupancy, energy root-mean-square deviation (RMSD), root-mean-square fluctuation (RMSF) were analysed by Gromacs built-in tools and our inhouse scripts (Table 2). The hydrogen-bond occupancies are shown in Fig. 3C, D, Tables S4 and S5 in the ESI.† OriginPro 2021 was used to analyse the hydrogen bond, energy, RMSD, RMSF data (Fig. 6 and 7).

**Table 2:** The average values with standard deviations of RMSD of hACE2, spikes and spike-hACE2 complexes, distances between the spikes and hACE2, number of hydrogen bonds, interaction energy, van der Waals energy, electrostatic energy and total energy between hACE2 and spikes for the last 25 ns of 150 ns simulations

		RMSD (nm)	Distance (nm)	Number of hydrogen bonds	Interaction energy (KJ mol^−1^)	Van der Waal energy (KJ mol^−1^)	Electrostatic energy (KJ mol^−1^)	Total energy (KJ mol^−1^)
WT spike	hACE2	0.39 ± 0.06	0.17 ± 0.02	9 ± 5	−951 ± 387	−543 ± 76	−2811 ± 256	−3174 ± 219
	Spike	0.67 ± 0.07						
	Complex	2.25 ± 0.05						
D614G mutant spike	hACE2	0.34 ± 0.02	0.18 ± 0.02	4 ± 3	−440 ± 211	−209 ± 25	−2609 ± 283	−2799 ± 276
	Spike	0.64 ± 0.08						
	Complex	1.46 ± 0.11						

### Free-energy calculations

The molecular mechanics Poisson–Boltzmann surface area (MMPBSA) was used to calculate the binding free energy. The binding free-energy calculations were made according to the following equation:Δ*G*_Binding_ = *G*_complex_ − (*G*_protein_ + *G*_ligand_)

## Results

### Binding processes of the SARS-CoV-2 WT and D614G mutant spikes with hACE2

Fig. 3 shows the binding processes of initial 20 ns and hydrogen-bond occupancies of interacting residues between hACE2 and B chains of spikes during the 150 ns MD simulations.

According to Fig. 3A, hACE2 has not contacted with the B chain of WT spike at 2 ns. It can be seen that 3 residues (Gln498, Pro499, Thr500) of the B chain contact with 3 residues (Gln86, Glu87, Gln89) of hACE2 at 5 ns, generating 1 hydrogen bond and 10 non-bonded interactions as well as 183 and 174 Å^2^ interface areas in the WT spike and hACE2 respectively. When the simulation time reaches 10 ns, 4 residues of the B chain of WT spike and 4 residues of hACE2 form 1 hydrogen bond and 14 non-bonded interactions. The interface areas of the B chain and hACE2 are 178 and 178 Å^2^. It is worth noting that the hydrogen-bond occupancies of Glu87-Gln498 formed at 5 ns and Gln89-Pro499 formed at 10 ns (Fig. S2 and S4 in the ESI†) are only 5% and 1% (Fig. 3C) in the 150 ns MD simulation, demonstrating that the hydrogen bonds formed are unstable. At 20 ns, 2 hydrogen bonds and 12 non-bonded interactions have formed among 3 residues of the B chain of WT spike and 3 residues of hACE2, generating 118 and 123 Å^2^ interface areas in the WT Spike B chain and hACE2, respectively. At this moment, a relatively stable hydrogen bond is observed between Gln89 and Thr500, with a hydrogen-bond occupancy of 10%. It can be noted that the contacts formed by 3 residues of hACE2 (Gln86, Glu87, Gln89) and 3 residues of WT spike (Gln498, Pro499, Thr500) at 5 ns transform into 3 other residues of WT spike (Thr500, Gly502, Tyr505) at 20 ns. The non-bonded interactions between Gln86 of hACE2 and Thr500 of WT spike change to Gln89 of hACE2 and Thr500 of WT spike. At 50 ns, there exist 16 residues and 687 Å^2^ interface area of the B chain of WT spike in contact with the 14 residues and 725 Å^2^ interface area of hACE2. The number of hydrogen bonds and non-bonded interactions are 8 and 86. From 50 ns to 150 ns, the interface areas of the B chain of WT spike and hACE2 have not changed significantly. During the 150 ns MD simulation, we observed two stable hydrogen bonds formed between Glu87 of hACE2 and Arg403, Gly496 of WT spike, with the hydrogen-bond occupancies of 79% and 76% respectively, which may enhance the interaction between WT spike and hACE2. The interacting residues between hACE2 and the WT spike at 2 ns, 5 ns, 10 ns, 20 ns, 50 ns, 75 ns, 100 ns and 150 ns are shown in Fig. S2 in the ESI.†

However, the binding process of the D614G mutant with hACE2 is quite different from the process described above. We noticed that the Gln493 of the B chain of D614G mutant spike forms a hydrogen bond and 2 non-bonded interactions with Ser19 of hACE2 (Fig. S3 and S5 in the ESI†), generating 106 Å^2^ and 118 Å^2^ interface areas of B chain and hACE2 respectively. According to the hydrogen-bond occupancies shown in Fig. 3D, hydrogen-bond occupancy of Ser19 and Gln493 is the largest, which indicates that the contact between D614G and hACE2 is stable at 2 ns. At 5 ns, 6 amino acid residues of the B chain of D614G mutant spike are in contact with 4 residues of hACE2. The interface areas are 231 Å^2^ and 276 Å^2^ for the B chain of D614G mutant spike and hACE2. In addition, the number of hydrogen bonds and non-bonded interactions increase to 5 and 36. Different from the unstable interaction formed initially between hACE2 and the WT spike, the 5 hydrogen bonds formed by 3 residues of hACE2 and 4 residues of D614G mutant spike have middle to good hydrogen-bond occupancy at 5 ns (Ser19 and Ser494 form two hydrogen bonds) as well as a stable interaction. At 10 ns, the initial interaction area, the number of hydrogen bonds and non-bonded interactions between the B chain of D614G mutant spike and hACE2 continue to increase. The 11 amino acid residues of the D614G mutant spike and 5 residues of hACE2 are connected by 5 hydrogen bonds and 50 non-bonded interactions, forming 358 Å^2^ and 434 Å^2^ interface areas of the B chain and hACE2 respectively. From 10 ns to 20 ns, the interface areas, number of interface residues, hydrogen bonds and non-bonded interactions of the D614G mutant with hACE2 show no significant increase. At 150 ns, 12 residues with 514 Å^2^ interface area of the B chain of D614G mutant spike are in contact with 9 residues with 542 Å^2^ area of hACE2, generating 2 hydrogen bonds and 67 non-bonded interactions between them. We further analysed the hydrogen-bond occupancy between D614G and hACE2, and found that more than 25% occupancy of hydrogen-bond formed during the initial 50 ns, demonstrating that the initial contact between D614G and hACE2 is quite stable. The interacting residues between hACE2 and the D614G mutant spike at 2 ns, 5 ns, 10 ns, 20 ns, 50 ns, 75 ns, 100 ns and 150 ns are shown in Fig. S3 in the ESI.†

RBD can usually be divided into three regions according to the characteristics of the binding process, including the polar contact regions C1 (Glu 471-Tyr 489) and C3 (Gly 446-Tyr 451, Tyr 495-Gly 502) at both ends of the interaction area, and the hydrophobic region C2 in the middle (Kys 417, Leu 452-Phe 456, Phe 490-Ser494).^53^ It has been reported C3 of WT RBD and hACE2 interacts at the beginning, which is consistent with the interaction area of WT spike and hACE2 (Thr 500, Pro 499, Gln 498) in our study. However, the process of D614G mutant is different. The initial contact between hACE2 and D614G mutant spike occurs in the C2 region (Gln 493), and then hACE2 interacts with C3 quickly. We also analyzed the interactions between hACE2 and the C1–C3 regions of RBD through distances between the residue centers after the 150 ns MD simulations. The distances between the 16 residues of WT spike RBD and 10 residues of hACE2 are less than 1.1 nm. The residues of WT RBD are evenly distributed in C1–C3 regions except for Val445 and Arg403 (Gln485, Phe486, Asn487 and Tyr489 in C1 regions, Tyr453, Leu455, Phe456 and Ser494 in C2 regions, Gly446, Gly447, Asn448, Tyr449, Tyr 495 and Gly496 in C3 regions). Compared with WT spike, the center distances of only 10 residues of D614G mutant and 8 residues of hACE2 are less than 1.1 nm. The residues of D614G mutant RBD are mainly distributed in C2 region except for Tyr505, 5 residues in C2 region (Lys417, Tyr453, Leu455, Gln493, Ser494), 3 residues in C3 region (Tyr449, Tyr495, Gly496), and 1 residue in C1 region (Ala475). The less contact regions in C1 and C3 may be the reason for the decrease of interaction area between hACE2 and D614G mutant spike.

### The Architectures and Electrostatic Surface Potentials of the SARS-CoV-2 WT and D614G Mutant Spikes after the 150 ns Simulations.

Subsequently, we analyzed the RBD structure of the WT spike after the 150 ns simulation. We found that RBD rotates outward by 3°, and the overall length of the RBD decreases from 88 Å to 78 Å (Fig. 4A). Contrarily to the results of the WT spike, RBD of the D614G mutant rotates inward by 16°, and the overall length increases from the initial 88 Å to 96 Å after 150 ns (Fig. 4C). By comparing the structural changes of the RBD structures for the two spikes, we noticed that RBD of the D614G mutant spike expands stretching upward while RBD of the WT spike contracts downwards during the binding processes with hACE2. In addition, we investigated the structural changes of the NTD structures after the 150 ns simulations. From the side views of the NTD structures (Fig. 4A and C), we found that the NTD structures of WT spike and D614G mutant spike have no obvious expansion or contraction over the simulation time. From the top views of the NTD structures (Fig. 4B), it can be seen that the NTD structures in B chain, C chain and D chain rotate 22° clockwise, 6° clockwise and 12° counterclockwise respectively. According to Fig. 4D, the NTD structures in B chain, C chain and D chain rotate 16° clockwise, 10° clockwise and 3° counterclockwise respectively. In order to quantify the structural changes of NTD structures, we calculated the RMSD values of NTD structures after the 150 ns MD simulations. The RMSD values of NTD structures in B chain, C chain and D chain of WT spike are 1.567 nm, 1.251 nm and 1.04 nm respectively, while the values of NTD structures in B chain, C chain and D chain of D614G spike are 1.114 nm, 1.209 nm and 1.394 nm respectively. In order to carefully examine the expansion or contraction of NTD structures, the distances between the Ser255 of NTD structures in different spike chains (Ser255.B-Ser255.C: BC, Ser255.C-Ser255.D: CD, Ser255.D -Ser255.B: DB) were measured. The Ser255 is on the surface of the spike and locates close to the edge of the NTD. The results show that the distances between two specific Ser255 residues of the WT spike change from BC-117 Å, CD-122 Å, and DB-115 Å (Fig. S6A in the ESI†) to BC-137 Å, CD-116 Å, and DB-98 Å, respectively. And the inner area of the triangle reduces from 6017 Å^2^ to 5581 Å^2^. In addition, the distances between two specific Ser255 residues of the D614G mutant alter from BC-115 Å, CD-115 Å, and DB-115 Å (Fig. S6B in the ESI†) to BC-126 Å, CD-130 Å, and DB-114 Å, respectively. The inner area of the triangle reduces from 5726 Å^2^ to 6528 Å^2^.

We further analysed changes of the electrostatic surface potentials of WT and D614G mutant spikes with hACE2 (Fig. 5). In Fig. 5, RBD is observed to have higher negative charges, and spike has relatively equal negative and positive electrostatic potentials.

The sum formal charges (SFCs) of hACE2, WT spike and D614G mutant spike were analysed by PyMOL. The SFC of hACE2 is −28 kT/e before the simulation, and the SFCs of hACE2 interacting with WT and D614G spikes are −27 kT/e after the 150 ns MD simulations, demonstrating that there is no significant difference in the electrostatic potential of hACE2 during the binding processes with two spikes. The SFCs of WT spike are −8 kT/e and −6 kT/e before and after the 150 ns MD simulation, respectively. In terms of the D614G mutant spike, the SFCs are −5 kT/e and −3 kT/e before and after 150 ns MD, respectively. According to the results of SFCs, the D614G mutation could reduce the negative electrostatic potential of spike, but not affect the change of electrostatic potential during the contact.

### Analyses of RMSD, RMSF, distances, hydrogen bonds and interaction energy

The average values with standard deviations of RMSD of spikes, hACE2 and their complexes for the last 25 ns of 150 ns simulations are summarized in Table 2. The RMSD curves for the last 25 ns of 150 ns simulations can be found in Fig. S7 in the ESI.†Fig. 6 shows the evolutions of the RMSD values for the WT spike, hACE2 and their complex (Fig. 6A), as well as the D614G mutant spike, hACE2 and their complex (Fig. 6B) over the whole 150 ns simulations. It can be noticed that the RMSD value of WT spike with hACE2 reaches a stable stage after 25 ns, while the value of D614 mutant spike with hACE2 reaches a stable stage after 100 ns. According to Table 2, the RMSD values of hACE2, the WT spike and their complex stabilize at 0.39 ± 0.06 nm, 0.67 ± 0.07 nm, and 2.25 ± 0.05 nm, respectively. Meanwhile, the RMSD values of hACE2, the D614G mutant spike and their complex stabilize at 0.34 ± 0.02 nm, 0.64 ± 0.08 nm and 1.46 ± 0.11 nm, respectively. In addition, no significant difference is observed by comparing the RMSD values of hACE2 in contact with the two different spikes. According to the RMSD values of the two spikes, the D614G mutant spike shows larger fluctuation than the WT spike, indicating the worse structural stability of the D614G mutant spike. However, the RMSD value of the D614G mutant spike-hACE2 complex has lower fluctuation (better structural stability) than the WT spike-hACE2 complex, demonstrating that the D614G mutant spike possesses stronger binding ability with hACE2 than the WT spike.

Subsequently, we analysed the RMSF values for residues in the RBD regions of the B chains of WT and D614G mutant spikes (Fig. 7A). It shows that the RMSF values of residues in RBD region of the D614G mutant are generally larger than those of the WT spike. Especially, the RMSF values of 450–492 residues in RBD of the D614G mutant spike are significantly larger than those of the corresponding residues in the WT spike. For the WT spike, 356–378 residues in RBD fluctuate more significantly than their counterparts in the D614G mutant spike.

From Table 2, it can also be noticed that there are no obvious differences in the distances between the spike and hACE2 for the WT (0.17 ± 0.02 nm) and D614G mutant (0.18 ± 0.02 nm) spikes. The average interaction energy between the WT spike and hACE2 is −951 ± 387 KJ mol^−1^, which is lower than the value of −400 ± 211 KJ mol^−1^ between the D614 mutant and hACE2. In addition, we generated the van der Waals energy, electrostatic energy, and total energy between hACE2 and spikes through MMPBSA. The results are consistent with the results of interaction energy. The energy values between WT spike and hACE2 are lower (van der Waals energy −543 ± 76 KJ mol^−1^, electric energy −2811 ± 256 KJ mol^−1^ and total energy −3174 ± 219 KJ mol^−1^) than those between D614G and hACE2 (van der Waals energy −209 ± 25 KJ mol^−1^, electric energy −2609 ± 283 KJ mol^−1^ and total energy −2799 ± 276 KJ mol^−1^).The number of hydrogen bonds and interaction energy between hACE2 and the spikes for the last 25 ns of 150 ns simulations are plotted in Fig. S8 in the ESI.† At the same time, the average number of hydrogen bonds between WT spike and hACE2 (9 ± 5) is higher than the value between D614 mutant and hACE2 (4 ± 3). By comparing the interaction energy and number of hydrogen bonds of the WT and D614G mutant spikes with hACE2, we can find that the WT spike possesses higher binding strength to hACE2 than the D614G mutant spike. The standard deviations of RMSD of hACE2, the spikes and spike-hACE2 complexes, distances between the spikes and hACE2, number of hydrogen bonds and interaction energy between hACE2 and the spikes for the last 25 ns of 150 ns simulations are summarized in Table S6 in the ESI.†

**Fig. 8:**
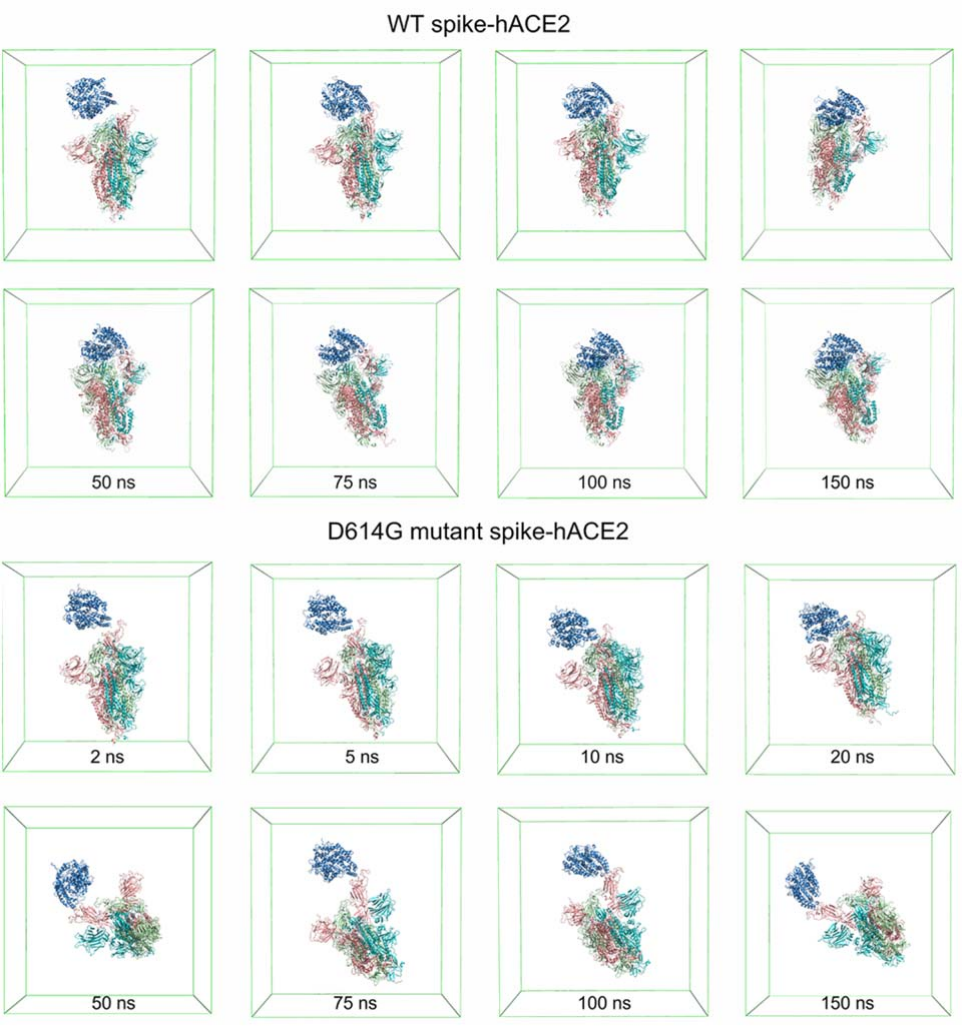
Molecular conformations of WT spike-hACE2 and D614G mutant spike-hACE2 complexes in the simulation boxes at different time.

From Fig. 7B, it can be noticed that the distance between the WT spike and hACE2 decreases to less than 0.5 nm after 2.75 ns, and consecutively reaches a distance without much fluctuation (0.17 ± 0.02 nm) after 5.25 ns. For the D614G mutant and hACE2, the distance between them decreases to less than 0.5 nm after 1.35 ns, and then reaches a distance without much fluctuation (0.17 ± 0.02 nm) after 1.52 ns. Based on these findings, it can be known that the D614G mutant spike moves towards the hACE2 faster than the WT spike. Fig. 7D shows the interaction energy of the D614G mutant and WT spikes during the whole 150 ns simulations. The interaction energy between the D614G mutant spike and hACE2 is lower than that between WT spike and hACE2 for the last 25 ns of 150 ns simulations. Conversely, the interaction energy between the D614G mutant spike and hACE2 becomes higher than that between WT spike and hACE2 after 25 ns. The change of number of hydrogen bonds shows the same tendency to the interaction energy (Fig. 7C). The results further suggest that the binding strength of WT spike-hACE2 is higher than the D614G mutant spike-hACE2 complex. The conformational changes of WT spike-hACE2 and D614G mutant spike-hACE2 complexes with simulation boxes plotted during the whole simulation time are shown in Fig. 8.

## Discussion

In this study, we performed the all-atom MD simulations to investigate the effects of single-point D614G mutation on the binding process between the SARS-CoV-2 spike and hACE2. Based on the simulation results, we find that the WT spike-hACE2 complex has the lower interaction energy than that of the D614G mutant spike-hACE2 complex. In addition, the WT spike and hACE2 have the larger interface areas (637 Å^2^ of the WT spike and 704 Å^2^ of hACE2) than their counterparts (514 Å^2^ of the D614G mutant spike and 542 Å^2^ of hACE2) after the 150 ns simulations. Luban and co-worker have calculated the equilibrium differentiation constants (*K*_D_) of D614G mutant and WT spikes with hACE2. At 25 °C, the *K*_D_ of D614G mutant spike and hACE is 5.7 folds to WT spike and hACE2 (*K*_D_ of D614G mutant spike and hACE2 is 7.97 nM, *K*_D_ of WT spike and hACE2 is 1.38 nM), and 5.3 folds at 37 °C^25^ (*K*_D_ of D614G mutant spike and hACE2 is 3.76 nM, *K*_D_ of WT spike and hACE2 is 0.71 nM), which may be caused by the low binding strength of hACE2 and D614G mutant spike. We hypothesize that the higher infectivity of the D614G mutant is independent of the binding strength of spike to hACE2, which is consistent with previous studies.^32^ Based on the structural analyses over the 150 ns simulations, we propose a rational mechanism that the impact of D614G mutation on the infectivity is due to the corresponding conformational transition. Xu and co-worker have reported that the conformation of the spike could transit from the pre-fusion state to the post-fusion state. The spike was in a tightly closed state with inactivated FP in the absence of the hACE2 but shifted to an open state after the binding of hACE2 and RBD. The untwist/downward-shift movement of the S1 subunit led to unpacked/activated FPs and an up RBD.^27^ As we show, the RBD of the D614G mutant spike extends inwards and upwards, making the mutant spike more likely to contact hACE2. The research of Chen and co-worker have explained the reason that RBD of D614G mutant spike is more likely to open.^54^ RBD can interact with disulfide-containing segment immediately downstream of the fusion peptide (residues 828 to 853, which Chen and co-worker named FPPR) through CTD1 mediated, and formation disorders of FPPR could cause the up and close of RDB. When D614 is mutated to G614, the salt bridge between D614 and K854 disappears, this change could perturb the structured FPPR to affect the open of RBD and lead to more likely binding of spike with hACE2.^55^ As same as RBD, NTD also exhibits flexibility, and the conformational dynamics is mainly reflected between the close and open states.^54^ The conformational of NTD is related to the open of RBD and the untwisting of S1. When NTD moves downward/outward, it is conducive to the open of RBD and could release the protomer interaction strength between S1 and S2.^31^ By analysing the MD results, the NTD of the D614G mutant spike extends more outwards than that of the WT spike, causing S1 domain away from the S2 and the D614G mutant spike more infectious.

By visualizing the binding processes, we can clearly observe that the interaction areas of the two spikes with hACE2 are completely different. For the D614G mutant spike, the initial interaction area Gln493 of B chain forms hydrogen bonds and non-bonded interactions with Ser19 of hACE2 at 2 ns. Later, hydrogen bonds and van der Waals forces increase within the initial interaction area, and thus leading to the increase of interaction area. However, after the initial contact between the WT spike and hACE2 (Gln498, Pro499, Thr500 of the WT spike B chain contact with 3 residues Gln86, Glu87, Gln89), the interaction area changes. We suggest that the initial interaction area of the D614G mutant and hACE2 is a relatively stable interaction area while the initial interaction area of the WT spike and hACE2 is an inferior stability interaction area. By analyzing the changes of distances between the spikes and hACE2, interaction energy and hydrogen bonds, we can find that the binding speed of the D614G mutant spike and hACE2 is relatively faster. The time for WT spike contacting hACE2 is about 2.75 ns, which is twice the contact time of D614G mutant spike with hACE2. Meanwhile, the time for the WT spike-hACE2 complex reaching the stable distance is 5.25 ns, which is triple time of D614G mutant spike-hACE2 complex. We propose such a slower contact process could also influence the probability of the new coronavirus to invade human cells.

## Conclusions

In this work, the differences of binding processes of the D614G mutant and WT spikes with hACE2 have been carefully compared. The results demonstrate that the initial interaction area of the D614G mutant with hACE2 is relatively stable while the initial interaction area of the WT spike with hACE2 is an inferior stability interaction area. Therefore, the WT spike and hACE2 represent a changing behavior of contact areas. At the same time, we observe that WT spike contacts hACE2 slower than the D614G mutant spike. According to the conformational changes of the D614G mutant and WT spikes after the 150 ns MD simulations, the RBD of the D614G mutant spike is more likely to contact hACE2 and the FP is easier to be unpacked/activated because of the open NTD. Our work provides the underlying mechanism for the high infectivity of the D614G mutant and hopefully could offer some guidance on intervention designs in future.

## Author contributions

Chengcheng Shi: writing-original draft, data curation, formal analysis. Yanqi Jiao: writing-original draft. Chao Yang: conceptualization, supervision. Yao Sun: conceptualization, writing-review & editing, validation, supervision.

## Conflicts of interest

There are no conflicts to declare.

## Supplementary Material

RA-013-D3RA00198A-s001
