# Supplementary material for: The influence of single-point mutation D614G on the binding process between human angiotensin-converting enzyme 2 and the SARS-CoV-2 spike protein-an atomistic simulation study
Source: RSC Adv. 2023 Mar 28;13(15):9800–10. doi: 10.1039/d3ra00198a (PMC10044093; doi:10.1039/d3ra00198a)
Supplement: RA-013-D3RA00198A-s001 [file RA-013-D3RA00198A-s001.pdf]

Electronic Supplementary Material (ESI) for RSC Advances.
This journal is © The Royal Society of Chemistry 2023

Supplementary information

The Influence of Single-Point Mutation D614G on the Binding Process Between Human Angiotensin-Converting Enzyme 2 and the SARS-CoV-2 Spike Protein-An Atomistic Simulation Study

Chengcheng Shi,[b] Yanqi Jiao,[a] Chao Yang [b,] and Yao Sun [a,*]*

[a] *School of Science, Harbin Institute of Technology (Shenzhen), Shenzhen 518055, China.*

[b] *State Key Lab of Urban Water Resource and Environment, School of Science, Harbin Institute of Technology (Shenzhen), Shenzhen 518055, China.*

*) Corresponding authors:

Dr. Yao Sun (sunyao0819@hit.edu.cn)

Dr. Chao Yang (xyyang@hit.edu.cn)

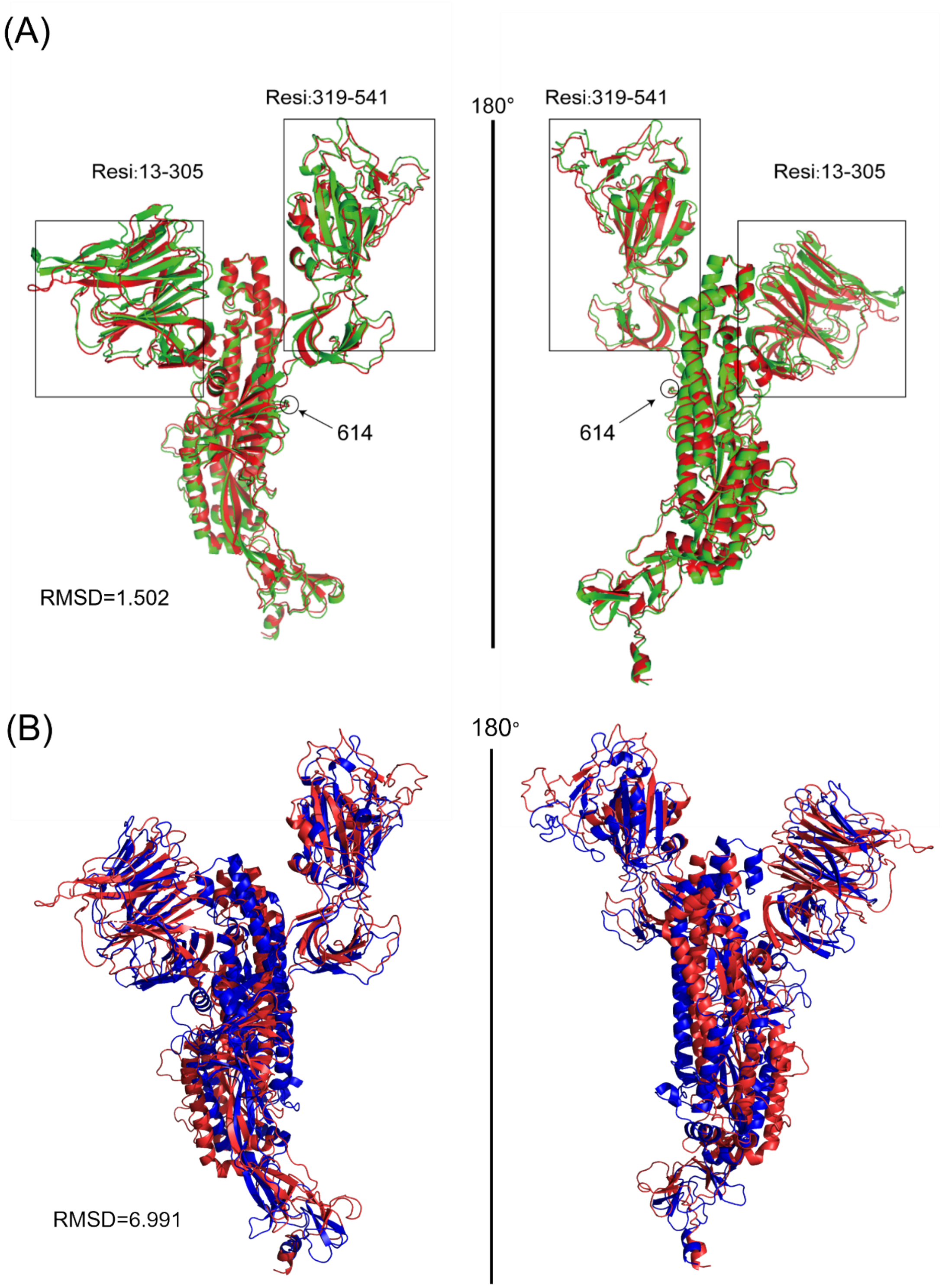

Figure S1. (A) The predicted single-chain (B chain) structural model of D614G mutant spike by AlphaFold2 which has the overall quality factor of 87.525 compared with the WT spike model (PDB ID :7DF4, B chain). The WT spike is colored in red while the predicted structure is colored in green. (B) The single-chain (B chain) structural model of D614G mutant spike by homologous modelling which has the overall quality factor of 62.332 compared with the WT spike model (B chain). The WT spike is colored in red while the homologous modelling is colored in green.

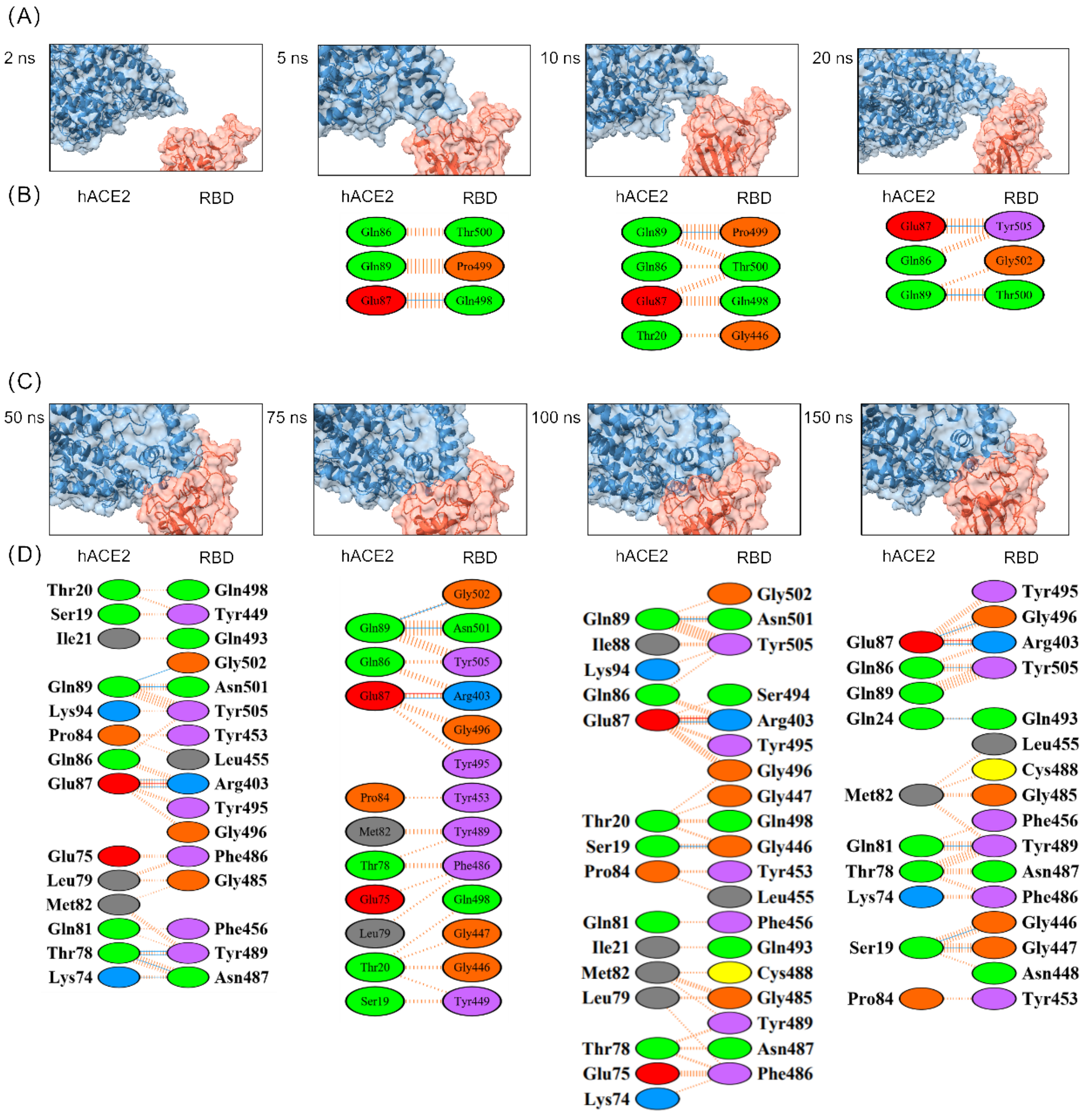

Figure S2. Binding process of the WT spike with hACE2 at 2 ns, 5ns, 10 ns, 20 ns, 50 ns, 75ns, 100 ns, 150 ns, (A) and (C) The binding interface, hACE2 is colored in steel blue and RBD is colored in red. (B) and (D) The interaction representation, which includes hydrogen (▬), salt bridges (▬), and non-bonded (▪ ▪ ▪) interactions. The A chain represents hACE2 and B chain represents WT RBD. Residue colors: positive (blue): H, K, R; negative (red): D, E; neutral (green): S, T, N, Q; aliphatic (grey): A, V, L, I, M; aromatic (purple): F, Y, W; Pro & Gly (orange): P, G.

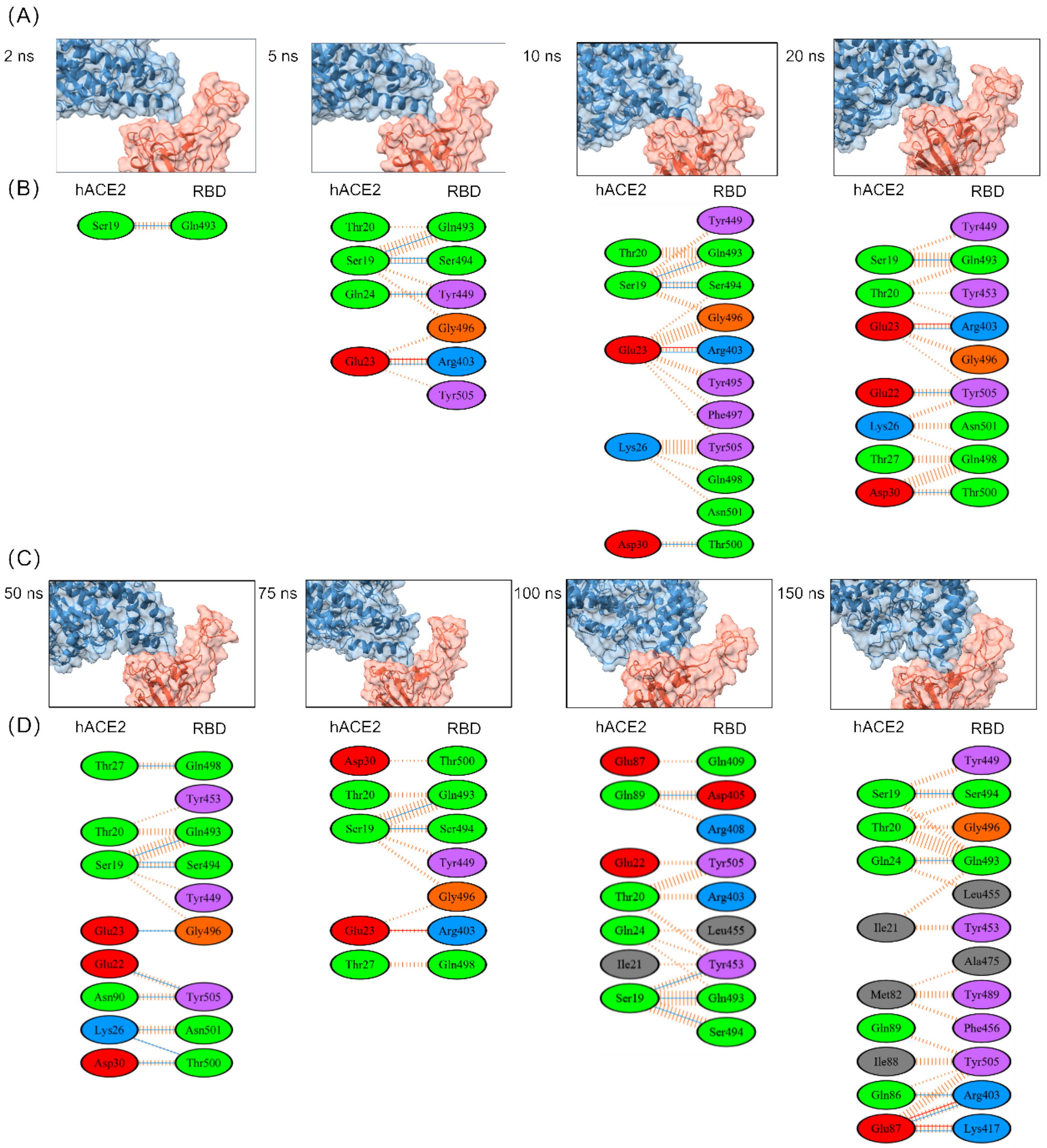

Figure S3. Binding process of the D614G mutant spike with hACE2 at 2 ns, 5ns, 10 ns, 20 ns, 50 ns, 75ns, 100 ns, 150 ns, (A) and (C) The binding interface, hACE2 is colored in steel blue and RBD is colored in red. (B) and (D) The interaction representation, which includes hydrogen ( ), salt bridges ( ), and non-bonded ( ) interactions. The A chain represents hACE2 and B chain represents WT RBD. Residue colors: positive (blue): H, K, R; negative (red): D, E; neutral (green): S, T, N, Q; aliphatic (grey): A, V, L, I, M; aromatic (purple): F, Y, W; Pro & Gly (orange): P, G.

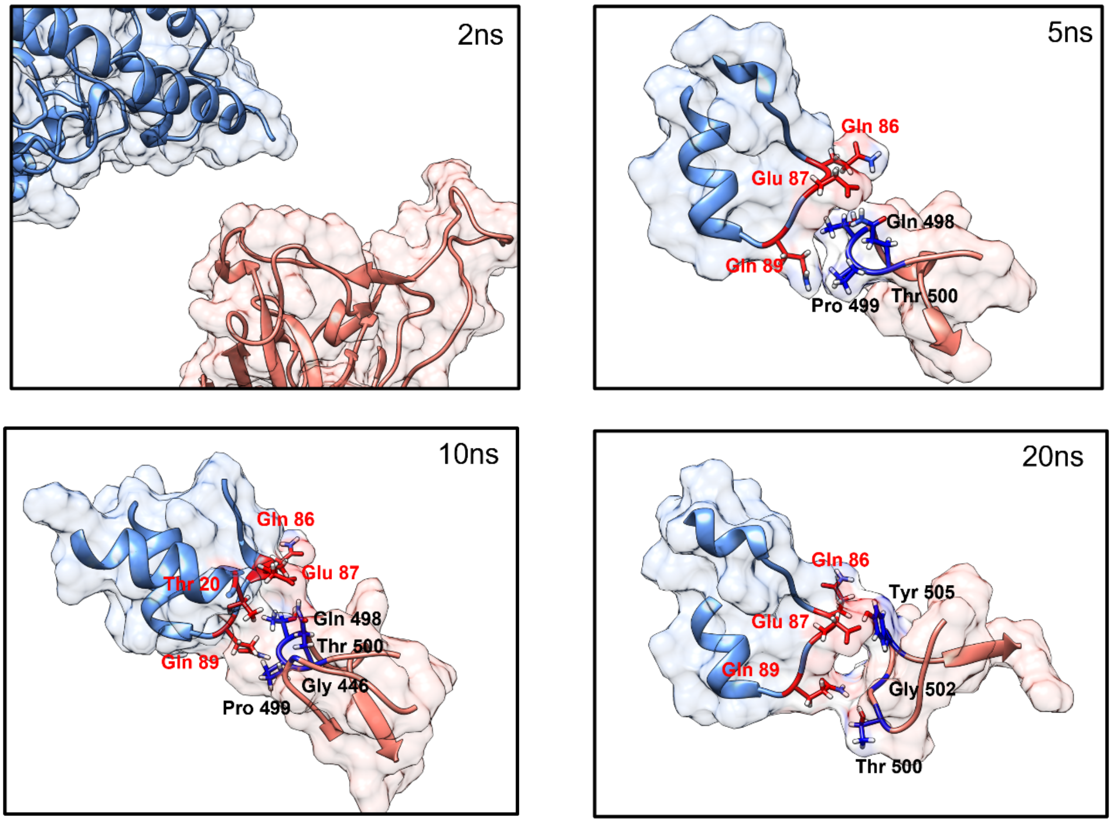

Figure S4. Binding processe of B chain of the SARS-CoV-2 WT spike with hACE2 at 2 ns, 5 ns, 10 ns and 20 ns. The spike B chain and hACE2 are colored in pink and steel blue respectively.

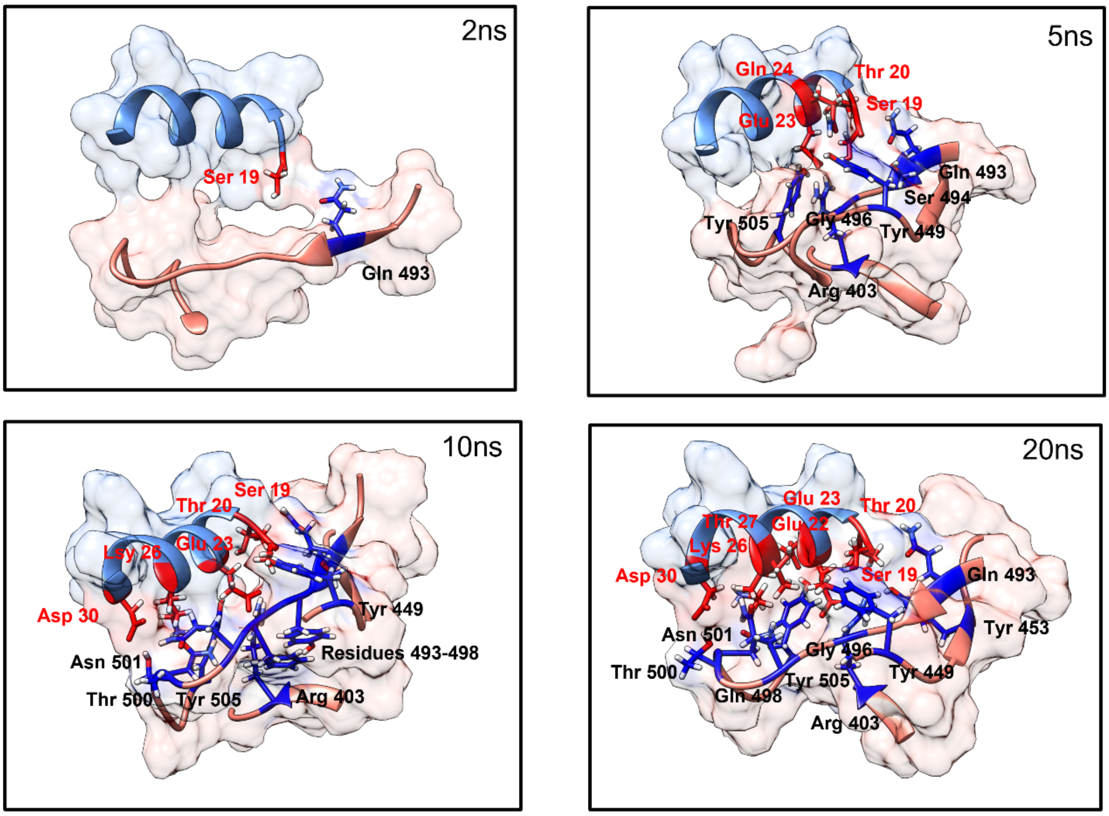

Figure S5. Binding process of B chain of the SARS-CoV-2 D614G mutant spike with hACE2 at 2 ns, 5 ns, 10 ns and 20 ns. The spike B chain and hACE2 are colored in pink and steel blue respectively.

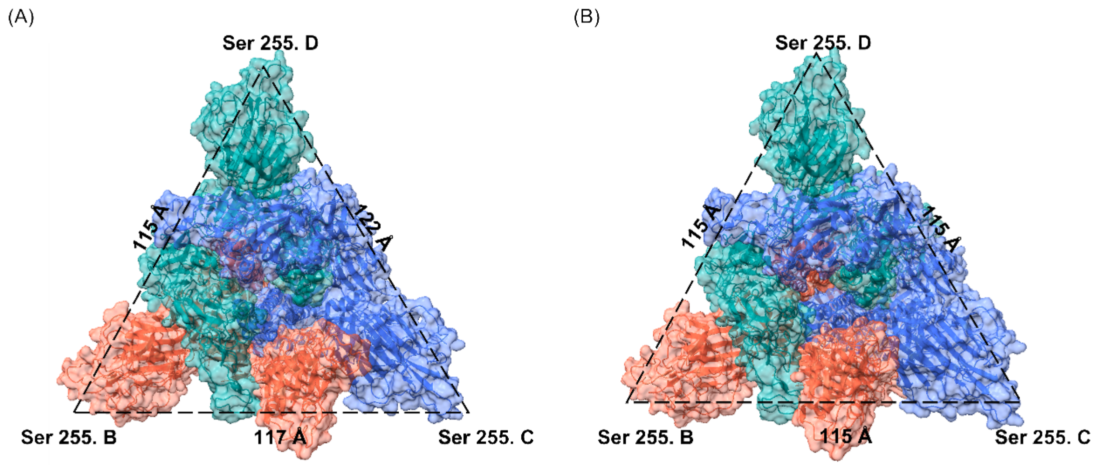

Figure S6. The architecture of the SARS-CoV-2 WT and D614G mutant spikes before the MD simulation, the spike chain B, chain C and chain D are colored in red, blue and cyan respectively. (A) top views of the WT spike, (B) top views of the D614G mutant spike.

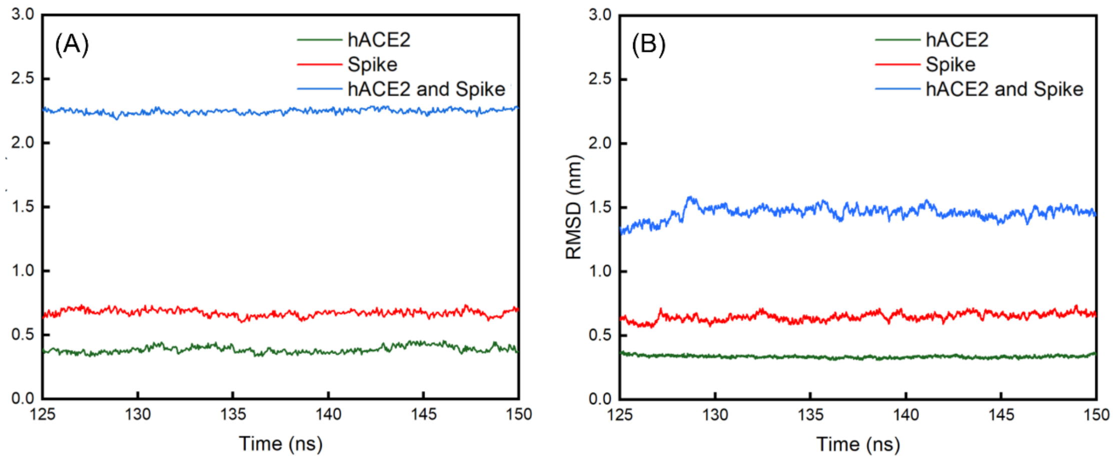

Figure S7. RMSD curves of the (a) WT spike, hACE2 and their complex and (b) D614G mutant spike, hACE2 and their complex for the last 25 ns of 150 ns simulations.

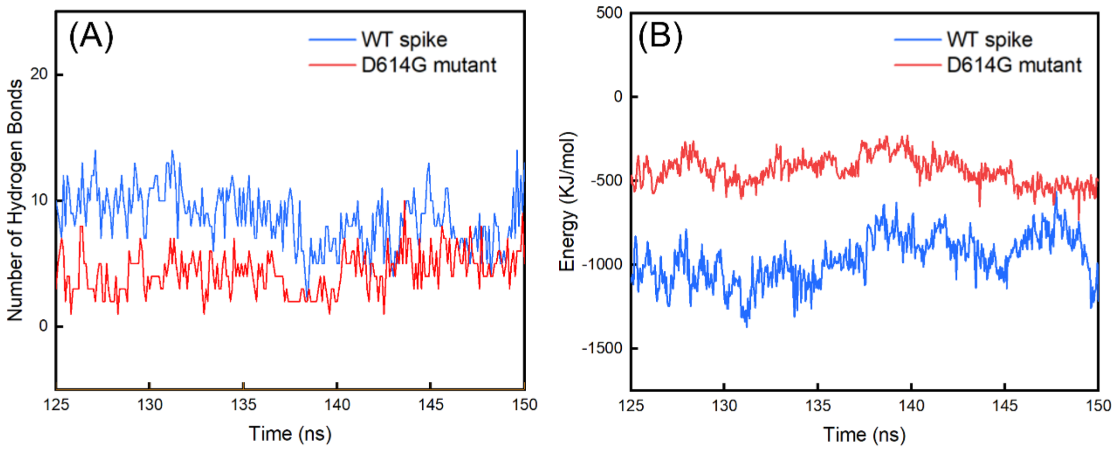

Figure S8. (a) Number of hydrogen bonds and (b) interaction energy between the spikes and hACE2 for the last 25 ns of the 150 ns simulations.

Table S1. Names and abbreviations of amino acids.

Full name	Three-letter abbreviation	One-letter abbreviation
Glycine	Gly	G
Alanine	Ala	A
Arginine	Arg	R
Proline	Pro	P
Serine	Ser	S
Glutamine	Gln	Q
Lysine	Lys	K
Aspartic acid	Asp	D
Glutamic acid	Glu	E
Asparagine	Asn	N
Threonine	Thr	T
Valine	Val	V
Isoleucine	Ile	I
Leucine	Leu	L

Table S2. Distances between residue centers of interaction of hACE2 and WT spike after the 150 ns MD simulation.

Residue of hACE2	Residue of WT spike	Distance between residue centers (nm)
Ser 19	Val 445	0.82708
Ser 19	Gly 446	0.72278
Ser 19	Gly 447	0.57547
Ser 19	Asn 448	0.60052
Ser 19	Tyr 449	0.59088
Ser 19	Ser 494	0.93896
Ser 19	Gly 496	0.84079
Thr 20	Gly 446	0.90055
Thr 20	Gly 447	0.76058
Thr 20	Gly 496	0.70315
Gln 24	Ser 494	0.96276
Glu 75	Phe 486	0.93780
Thr 78	Phe 486	0.79174
Thr 78	Asn 487	0.55766
Leu 79	Gly 485	0.84086
Leu 79	Phe 486	0.65684
Gln 81	Tyr 489	0.97779
Met 82	Leu 455	1.08876
Met 82	Phe 456	1.09066
Met 82	Tyr 489	0.89058
Pro 84	Tyr 453	0.98433
Pro 84	Leu 455	0.84575
Glu 87	Arg 403	1.06902
Glu 87	Tyr 495	1.09359
Glu 87	Gly 496	0.79744

Table S3. Distances between residue centers of interaction of hACE2 and D614G mutant spike after the 150 ns MD simulation.

Residue of hACE2	Residue of D614G mutant spike	Distance between residue centers (nm)
Ser 19	Tyr 449	0.64492
Ser 19	Gln 493	1.02630
Ser 19	Ser 494	0.59786
Thr 20	Gln 493	0.87034
Thr 20	Ser 494	0.51190
Thr 20	Tyr 495	0.78766
Thr 20	Gly 496	0.66417
Ile 21	Tyr 453	0.75903
Ile 21	Leu 455	0.94519
Ile 21	Gln 493	0.72020
Gln 24	Gln 493	1.00141
Met 82	Ala 475	1.05171
Glu 87	Lys 417	0.87540
Ile 88	Tyr 505	1.09979
Gln 89	Tyr 505	0.97617

Table S4. Hydrogen-bond occupancies of interacting residues between hACE2 and WT spike during the 150 ns MD simulation.

Residue of hACE2	Residue of WT spike	Hydrogen-bond occupancy
Ser 19	Gly 446	16.5%
Ser 19	Gly 447	2.9%
Gln 24	Gln 493	0.9%
Thr 78	Thr 487	46.9%
Thr 78	Thr 489	18.4%
Gln 81	Tyr 489	12.0%
Gln 86	Tyr 505	7.3%
Glu 87	Arg 403	78.7%
Glu 87	Gly 496	76.2%
Glu 87	Gln 498	5.1%
Glu 87	Tyr 505	1.7%
Gln 89	Pro 499	1.3%
Gln 89	Thr 500	9.9%
Gln 89	Asn 501	36.5%
Gln 89	Gly 502	43.6%

Table S5. Hydrogen-bond occupancies of interacting residues between hACE2 and D614G mutant spike during the 150 ns MD simulation.

Residue of hACE2	Residue of D614G mutant	Hydrogen-bond occupancy
Ser 19	Tyr 453	22.3%
Ser 19	Gln 493	60.8%
Ser 19	Ser 494	37.7%
Glu 22	Tyr 505	33.1%
Glu 23	Arg 403	25.7%
Glu 23	Gly 496	37.7%
Gln 24	Tyr 449	2.5%
Gln 24	Gln 493	15.4%
Lys 26	Thr 500	5.1%
Lys 26	Asn 501	20.5%
Thr 27	Gln 498	18.1%
Asp 30	Thr 500	45.9%
Gln 86	Arg 403	3.5%
Glu 87	Arg 403	13.1%
Glu 87	Lys 417	22.2%
Gln 89	Asp 405	9.9%
Asn 90	Thy 505	6.4%

Table S6. Standard deviations of RMSD of hACE2, the spikes and spike-hACE2 complexes, distances between the spikes and hACE2, number of hydrogen bonds and interaction energy, Van der Waal energy, electrostatic energy and total energy between hACE2 and the spikes for the last 25 ns of 150 ns simulations.

		**RMSD (nm)**	**Distance (nm)**	**Number of hydrogen bonds**	**Interaction energy (KJ/mol)**	**Van der Waal energy (KJ/mol)**	**Electrostatic energy (KJ/mol)**	**Total energy (KJ/mol)**
**WT spike**	hACE2	0.026	0.0069	2.24	143.95	53.74	190.52	154.68
	spike	0.025						
	complex	0.018						
**D614G mutant spike**	hACE2	0.010	0.0092	1.65	85.52	18.45	208.72	211.38
	spike	0.029						
	complex	0.049						
